# Symptom burden in chronic liver disease

**DOI:** 10.1093/gastro/goae078

**Published:** 2024-08-09

**Authors:** Ammar Hassan, Ivonne Hurtado Diaz De Leon, Elliot B Tapper

**Affiliations:** Division of Gastroenterology, Department of Internal Medicine, University of Michigan Health West, University of Michigan Medicine, Grand Rapids, MI, USA; Department of Gastroenterology, Instituto Nacional de Ciencias Médicas y Nutrición Salvador Zubirán, Mexico City, Mexico; Division of Gastroenterology and Hepatology, Department of Internal Medicine, University of Michigan, Ann Arbor, MI, USA

**Keywords:** Cirrhosis, sex, cramps, itch, falls, fatigue

## Abstract

Chronic liver disease (CLD) is a significant contributor to global mortality. For people who are living with CLD, however, there is a substantial and often overlooked burden of physical and psychological symptoms that significantly affect health-related quality of life. CLD frequently presents with a multitude of interrelated and intricate symptoms, including fatigue, pruritus, muscle cramps, sexual dysfunction, and falls. Increasingly, there is interest in studying and developing interventional strategies to provide a more global approach to managing these complex patients. Moreover, in addition to established guidelines for the management of conventional complications, such as ascites and hepatic encephalopathy, there have been efforts in developing evidence-based guidance for the treatment of the more subjective yet still problematic elements. This review will address the management of these less “classical” but nonetheless important symptoms.

## Introduction

Chronic liver disease (CLD) is a significant contributor to global mortality. It is estimated that liver disease accounts for approximately two million deaths annually, representing 4% of global mortality [1]. Moreover, cirrhosis is a major factor in global health loss, ranking as the 15th leading cause of disability-adjusted life years (DALYs) worldwide. Its most pronounced impact is seen among younger age groups, standing as the 12th leading cause of DALYs among individuals aged 25–49 years [1].

**Figure 1.**
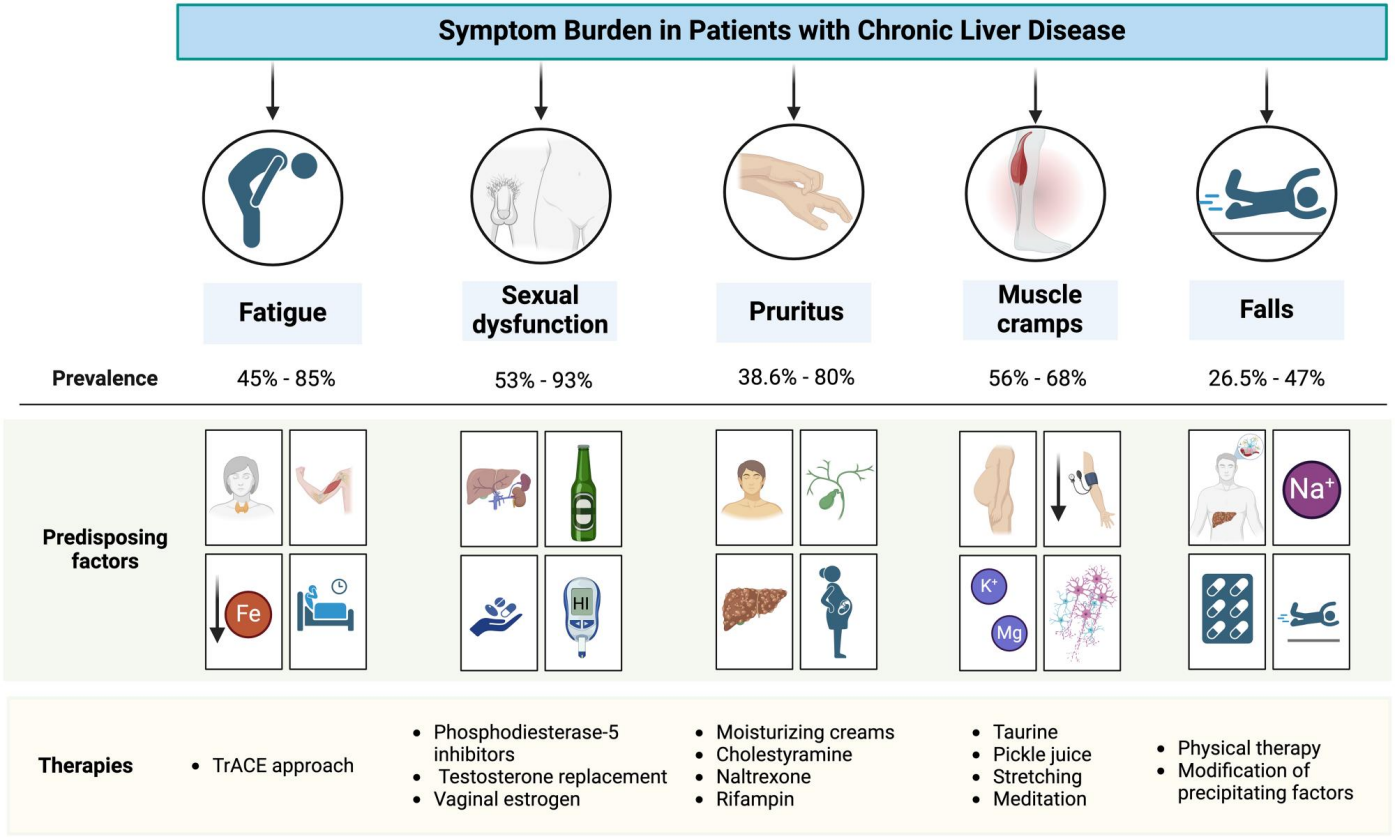
Common symptoms of CLD. We summarized the prevalence, precipitating factors, and interventions for common symptoms.

The spectrum of symptoms associated with CLD is broad and includes both physical and psychological aspects that significantly affect health-related quality of life (HRQoL) [2]. With the improved understanding and recognition of this domain in the care of patients with advanced liver disease, there has been growing interest in studying and developing interventional strategies to provide a more global approach to managing these complex patients [3]. Moreover, in addition to established guidelines for the management of conventional complications, such as ascites and hepatic encephalopathy, there have been efforts in developing evidence-based guidance for the treatment of more subjective yet still problematic elements, such as pain, fatigue, and muscle cramps [4]. CLD frequently presents with a multitude of interrelated and intricate symptoms, including fatigue, pruritus, muscle cramps, sexual dysfunction (SD), and falls, among others [5]. This review will address the management of these less “classical” but nonetheless important symptoms (Figure 1).

### Fatigue

Fatigue, a common and often debilitating nonspecific symptom [6, 7], appears to be one of the most frequently reported symptoms in patients affected by CLD [8, 9]. Estimates of the prevalence of fatigue vary across different studies and among different etiologies and severity levels of the disease, with an estimated prevalence in CLD ranging from 45% to 85% [10–12]. The pathophysiology of fatigue in CLD is multi-faceted, involving both peripheral and central mechanisms. Peripheral fatigue relates to neuromuscular dysfunction outside the central nervous system and may include sarcopenia, muscle weakness, and pain [10, 12]. Central fatigue arises from changes in neurotransmission within the brain, often linked with neuroinflammatory processes that affect mood and cognitive functions [12, 13]. The magnitude of fatigue symptoms also depends on the presence or absence of non-liver medical factors, such as endocrinopathies (hypothyroidism, adrenal insufficiency), anemia, vitamin deficiencies, malnutrition, among others [14, 15].

**Figure 2.**
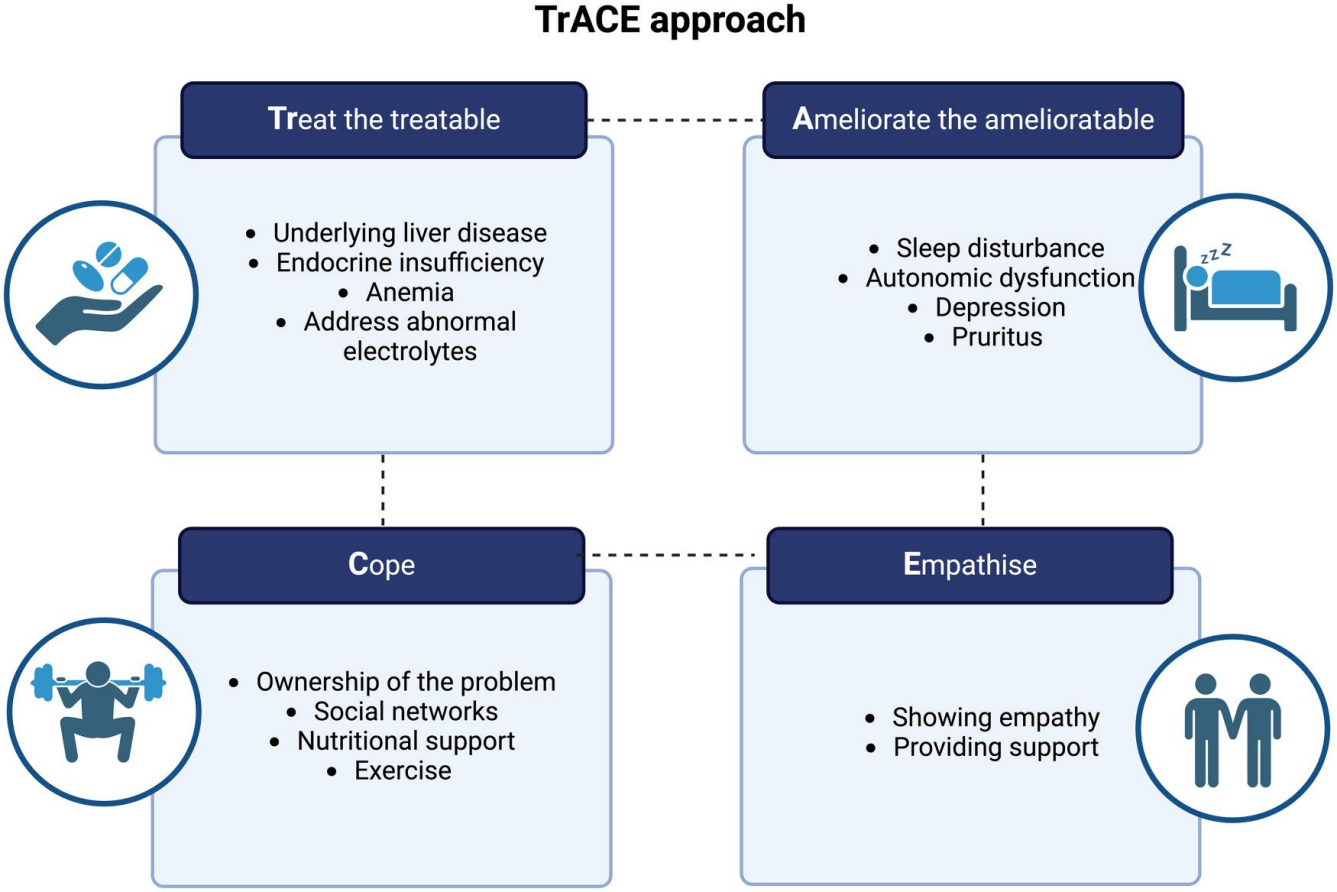
The TrACE approach to the symptom control. We adapted a method for the management of chronic disease by specifying a regimented approach to the bothersome symptoms of cirrhosis. This is particularly effective for vague or multifactorial symptoms, such as fatigue.

The management of fatigue represents a significant and complex clinical challenge. Currently, the primary approach is supportive, as there are no specific treatments available for this condition. We suggest the TrACE approach [12] (Figure 2):

1. Treat the treatable: address all symptoms and underlying conditions that may contribute to the onset or exacerbation of fatigue.
2. Ameliorate the ameliorable: The treatment plan should address conditions frequently associated with CLD, including sleep disturbance, depression, and vasomotor autonomic dysfunction.
3. Cope: assuming responsibility for addressing the issue and employing coping strategies. This entails the implementation of a structured daily schedule, the avoidance of shift work, the incorporation of exercise into daily routines, and the consumption of a diet that is appropriate for the clinical condition.
4. Empathise: show understanding and support.

### Sleep dysfunction

Closely linked to fatigue, sleep disorders are highly prevalent among individuals with CLD. Studies utilizing assessments like the Pittsburgh Sleep Quality Index have shown that the prevalence of these disturbances ranges from approximately 50% to 80% [16–20]. These disruptions correlate with decreased HRQoL and the presence of fatigue [18, 19]. In the context of CLD, pharmacological strategies could be considered for this particular symptom, although there is currently a lack of strong evidence to support this approach. In a double-blind, randomized controlled trial conducted in patients with cirrhosis and minimal HE, 35 patients with minimal HE and long-standing sleep difficulties were randomly assigned to receive either hydroxyzine 25 mg at bedtime or a placebo for a 10-day period. It was observed that hydroxyzine treatment resulted in subjective improvement in sleep in 40% of patients, in comparison to none in the placebo group. However, one patient experienced an acute episode of encephalopathy, which resolved upon discontinuation of hydroxyzine. Therefore, caution and close monitoring of patients are recommended when prescribing this medication [21]. Furthermore, an open-label study examined modafinil therapy in 21 patients with primary biliary cholangitis (PBC) experiencing significant daytime somnolence and fatigue [22]. Modafinil treatment was initiated at a dose of 100 mg/day and adjusted based on individual tolerance and response. After 2 months of treatment, modafinil significantly improved daytime somnolence (Epworth Sleepiness Scale scores: 15 ± 3 vs. 8 ± 6, *P *<* *0.0005) and fatigue severity (PBC-40 fatigue domain score: 46 ± 6 vs. 34 ± 12, *P *<* *0.0001). However, further investigation is warranted to provide any recommendations in this regard. Many patients with cirrhosis and portal hypertension may have covert hepatic encephalopathy. Treatment with lactulose, even as a time-limited trial (≥28 days) has the potential to improve sleep quality for many patients [23].

Ultimately, the intricate interactions between neural signaling pathways and the pathways involved in fatigue in CLD highlight a promising avenue for the development of targeted therapies, especially considering the current lack of available pharmacological treatments and the significant impact on the quality of life of those affected.

### Sexual dysfunction

SD can manifest with a wide range of symptoms, differing between men and women. In men, symptoms primarily fall into three categories: erectile dysfunction, low libido, and ejaculatory disorders [24]. For women, SD may include a lack of sexual desire, impaired arousal, inability to achieve orgasm, or pain during sexual activity that causes distress. To fulfill the diagnostic criteria for SD, the sexual issue must be recurrent or persistent and must cause personal distress or interpersonal difficulties.

In the context of CLD, the routes to SD are multifaceted and diverse. Hormonal imbalances brought on by cirrhosis often result in reproductive challenges and SD across genders [25]. For men, this typically involves an overproduction of estrogen due to portal hypertension and a decrease in testosterone from testicular dysfunction. Women, while also affected by hormonal fluctuations, mainly face disruptions in the hypothalamic-pituitary-gonadal axis, albeit with a subtler influence from portal hypertension compared to men [25]. Other potential causes of SD encompass various comorbid conditions, including diabetes mellitus, anxiety, and depression [25, 26]. Moreover, there is a clear association between the use of specific medications and the onset of SD as a side effect, which can play a role in initiating and worsening this condition in patients with CLD. Medications often prescribed for cirrhosis, such as spironolactone with its antiandrogenic effects, are linked to reduced libido and erectile dysfunction [25, 27]. Additionally, β-blockers, by causing vasodilation, suppressing the release of renin, and reducing the production of angiotensin II and aldosterone, can lead to diminished adrenergic activity from the sympathetic nervous system, which is another contributing factor to erectile dysfunction [27]. Furthermore, the progression of liver disease has a profound impact on sexual health, with those experiencing more advanced stages being increasingly affected [25]. Another factor contributing to the onset of SD is the underlying liver disease etiology [25]. For instance, there is a well-documented link between alcohol consumption and an increased likelihood of SD in both males and females [25, 28]. In men, alcohol’s direct harmful impact on the testes may also impair the secretion of gonadotropins, leading to testicular shrinkage and a consequent decrease in testosterone levels [26, 29].

SD is a condition commonly observed in individuals with CLD; however, it is often overlooked in clinical settings. In general, there is a notable deficiency in the evaluation of sexual health within primary care settings globally, which is influenced by the healthcare provider’s level of experience, their gender, and the age of the patients they treat [30]. The prevalence of erectile dysfunction alone has been reported to range from 53% to 93% [6]. In the case of women with CLD, the prevalence of SD has been reported to be as high as 77.8% [6]. In both genders, it has been observed that both the frequency of and interest in sexual activity have significantly declined, with 65.3% and 40.5% reporting decreases, respectively [31]. These changes negatively impact an individual’s emotional state, general health, and HRQoL [6, 25]. It is therefore imperative that patients with CLD are afforded the opportunity to undergo an assessment of their SD and to receive treatment where appropriate [4].

The assessment of SD encompasses a thorough physical examination, medical, and sexual history. For a comprehensive evaluation and diagnosis, the primary diagnostic instruments employed are the International Index of Erectile Function [32] for males and the Female Sexual Function Index [33] for females. These tools are recognized as reliable and objective measures for determining the presence and severity of SD.

Treatment of SD is complex and generally necessitates a multidisciplinary assessment. After identifying the type of SD, it is recommended to identify and eliminate predisposing factors, such as medications and substances, including alcohol, tobacco, and marijuana [34]. Additionally, it is important to optimize the management of associated medical conditions like depression and diabetes [25].

There is very limited data on the medical management of SD in patients with advanced liver disease [35]. Few studies have examined the effects of testosterone therapy in men with cirrhosis. Investigations into testosterone therapy in cirrhotic men have been limited by small patient populations, diverse patient demographics, and varying methods of medication administration. Additionally, these studies have mostly focused on alterations in muscle mass composition, such as sarcopenia, changes in serum albumin levels, and the reduction of gynecomastia, rather than on SD enhancement [29]. In a study involving 221 men with alcohol-related cirrhosis, 67% reported SD. Participants were skewedly randomized to receive either oral testosterone treatment (200 mg three times daily) or a placebo. Over a period of 30 months, improvements in sexual function were observed, yet there was no significant difference between the effects of oral testosterone treatment and placebo. This indicates that the enhancement in sexual health may be attributed to the reduction in alcohol consumption rather than to testosterone therapy [36]. For men experiencing erectile dysfunction, phosphodiesterase-5 (PDE5) inhibitors, such as sildenafil, tadalafil, avanafil, and vardenafil, can be considered as initial therapy. In a study aiming to evaluate the prevalence of erectile dysfunction in patients with CLD, along with associated factors and response to tadalafil therapy, data from 60 males with Child-Pugh scores between 5 and 10 and without overt HE were analyzed. Patients with erectile dysfunction were administered 10 mg of tadalafil for 4 weeks. The results showed a significant improvement in International Index of Erectile Function questionnaire scores after 4 weeks of tadalafil therapy, with no major serious adverse effects reported [37]. However, these medications are not recommended for patients categorized as Child-Turcotte-Pugh C [25]. In patients with less advanced liver disease, PDE5 inhibitors may be used, but with caution due to reduced drug clearance, often necessitating lower dosing [25]. In considering the treatment with PDE5 inhibitors, it is crucial to avoid side effects, such as hypotension. A pilot study investigating sildenafil’s impact on hemodynamics found that while the drug notably decreased mean arterial pressure due to its systemic vasodilatory properties, it did not have a significant effect on the hepatic venous pressure gradient [38]. There are no approved therapies for the pharmacological management of SD in women with cirrhosis [39]. However, for women reporting vaginal dryness, pain with intercourse, or frequent urinary tract infections, vaginal estrogen may be considered.

### Pruritus

Pruritus may occur in patients with CLD for a variety of reasons. While this symptom is not a particular cause of CLD, it is more frequently associated with cholestatic liver diseases where there are abnormalities in bile metabolism or biliary obstruction. Conditions, such as PBC, primary sclerosing cholangitis, chronic hepatitis, and intrahepatic cholestasis of pregnancy are notable examples where pruritus is a significant symptom [40]. The frequency of pruritus can be as high as 70%–80% in PBC patients, while in primary sclerosing cholangitis, it affects 20%–40% and tends to increase as the disease advances [40]. Though not as common in other CLDs, pruritus still affects up to 38.6% of individuals with cirrhosis [41]. Additionally, in individuals with chronic viral hepatitis, the prevalence of pruritus is reported to be between 35% and 44% [42]. Pruritus can severely diminish a patient’s HRQoL, frequently leading to mental health issues that can include sleep deprivation, increased risk of depression, and suicidal ideation [40].

Cholestatic pruritus may manifest across the body or in specific areas, particularly on the palms and soles. The intensity of this symptom does not correspond to the severity of the liver disease and can range widely. Typically, the itch worsens at night and may be soothed by cooler temperatures, providing relief [40].

The underlying mechanisms of pruritus in CLD remain elusive, but multiple theories have emerged. One suggests that the accumulation of bile acids acts as pruritogens, although the evidence is scant and controversial [43, 44]. Contrarily, pruritus sometimes subsides despite persistent cholestasis and elevated bile acid levels, and many patients with cholestasis do not experience pruritus despite high plasma bile acid levels [44]. Another hypothesis suggests that lysophosphatidic acid (LPA) may be involved in the pathogenesis of pruritus. LPA levels and autotaxin (ATX) activity, which converts lysophosphatidylcholine into LPA, are elevated in patients with cholestatic pruritus [45]. There is emerging evidence to suggest that endogenous opioids may play a role in the pathophysiology of cholestatic pruritus [44]. Primarily, this is due to several studies demonstrating a reduction in cholestatic pruritus in patients treated with opioid antagonists [46–51].

The initial approach to managing patients with cholestasis-associated pruritus involves addressing the underlying condition, if feasible. The severity of symptoms dictates the treatment approach. For mild symptoms, nonspecific measures like emollients may be sufficient [4]. However, for patients with moderate to severe pruritus unresponsive to the aforementioned measures, initiating pharmacologic therapy can be considered.

The primary strategy for addressing pruritus is to first identify and treat the underlying condition, whenever feasible. Alongside this treatment, first-line therapy includes symptomatic management, such as topical emollients, loose and soft textured clothing, and avoidance of exacerbating factors [52]. In cases where patients with moderate to severe pruritus do not find relief through general approaches, the next step is to consider pharmacological interventions.

- Bile acid sequestrants: essentially nonabsorbable basic polystyrenes, bile acid resins like cholestyramine, colestipol, and colesevalam, bind to anions, such as bile acids in the gut, thereby reducing bile acid levels by blocking their reabsorption [40]. Although there is currently insufficient evidence demonstrating the effectiveness of bile acid resins, they are commonly recommended as initial treatments due to their safety profile and the positive outcomes observed in clinical practice [4, 47, 53–55]. The initial dosage recommended for cholestyramine is 4 g per day. Depending on the patient’s individual needs and response, this dosage can be adjusted up to 16 g daily [40]. Nevertheless, due to the potential for impaired drug absorption, it is often recommended to space their administration apart from other medications [4, 44]. Among other potential adverse effects, gastrointestinal discomfort, headache, and constipation may occur [4, 44].
- Naltrexone: a μ-opioid antagonist, represents a potential alternative for treatment [4]. Several studies have demonstrated its efficacy in pruritus secondary to cholestasis [47, 48, 50, 51]. A starting dose of 12.5 mg of naltrexone once daily is recommended, with gradual dose escalation up to a maximum of 50 mg once daily, as tolerated and as needed. The objective of this recommendation is to mitigate the risk of opiate withdrawal reactions when initiating naltrexone therapy. Furthermore, it is advised to refrain from use in patients currently taking opioid-containing medications [4, 54]. Additionally, although relatively uncommon, hepatotoxicity associated with naltrexone may occur, highlighting the importance of meticulous monitoring of liver biochemistry [4].
- Rifampin: exhibits potent activity as an agonist of the pregnane X receptor (PXR) [44]. *In vitro* studies have shown that rifampicin inhibits ATX expression in human HepG2 hepatoma cells and in hepatoma cells overexpressing the PXR, but not in hepatoma cells in which PXR has been knocked down. This suggests that the antipruritic effect of rifampicin is mediated in part by a PXR-dependent transcriptional inhibition of ATX expression [56]. While evidence on the efficacy of rifampicin for the treatment of cholestatic pruritus is limited, several studies to date suggest its potential efficacy [57–61]. These findings were summarized in a meta-analysis designed to evaluate the safety and efficacy of rifampin in the treatment of pruritus associated with chronic cholestasis. The meta-analysis included five prospective, randomized, controlled, crossover studies involving 61 patients. Treatment with rifampin resulted in complete or partial resolution of pruritus in 77% of patients, compared with 20% treated with placebo. Seven percent of patients receiving rifampin experienced side effects, which resolved after discontinuation of the drug, and no cases of hepatotoxicity were reported [62]. Using doses ranging from 150 to 600 mg per day has been recognized as both an effective and safe approach [44]. Adverse effects associated with rifampin can range from mild to severe and include nausea, altered metabolism of other drugs, renal failure, and hepatotoxicity [44, 63].
- Antihistamines: although there is no specific recommendation to use antihistamines as part of pharmacological treatment, given that the itch in cholestasis is not histamine-driven, antihistamines may offer potential benefits. These benefits may be due to the sedative properties of antihistamines, which can enhance sleep quality and indirectly alleviate the perception of pruritus [4, 54, 63].

Additional therapies include Sertraline (an SSRI) which has also been shown to have a moderate antipruritic benefit in small randomized control trials [64]. Finally, although evidence is lacking for patients without cholestatic liver disease, pruritis among patients with cholestatic liver disease can often be improved with fibrate therapy. While bezafibrate is best studied, fenofibrate can be substituted but monitored for risks, such as myopathy.

### Muscle cramps

Muscle cramps are a common complaint in patients with cirrhosis, with an estimated prevalence ranging from 56% to 68% [6, 41]. The calves are the most common area affected, followed by the feet and thighs [65]. These symptoms disrupt sleep, impair mobility, and are a major factor in reduced HRQoL [41, 65]. The mechanism is complex and not fully understood, they are believed to result from a confluence of factors, including change in plasma volume, electrolyte imbalance, nerve abnormalities, and energy metabolism changes [65].

Currently, the options for successfully treating muscle cramps in individuals with cirrhosis are quite limited. The general approach to treatment typically focuses on restoring electrolyte balance and maintaining euvolemia [4, 65]. Pharmacologic options that have been studied and may be used include taurine, baclofen, vitamin E, branched-chain amino acids (BCAAs), and pickle juice [4]. In a study examining treatment options for muscle cramps in patients with cirrhosis, a systematic review was conducted to evaluate available therapeutic interventions. The review analyzed 24 publications, comprising seven randomized controlled trials and 17 prospective studies. The results indicated that taurine, methocarbamol, baclofen, and orphenadrine are viable and effective options for managing muscle cramps in cirrhosis patients. Additionally, l-carnitine, BCAAs, pregabalin, zinc, and vitamin D demonstrated promising outcomes. However, the findings regarding vitamin E were inconclusive. The study underscores the necessity for further well-designed randomized controlled trials to establish optimal treatment strategies [66].

- Pickle juice: based on current evidence, the use of pickle juice could be considered for the acute treatment of muscle cramps at the onset of symptoms. In a randomized trial involving 82 cirrhosis patients with frequent muscle cramps, pickle juice was evaluated for its efficacy in alleviating cramp severity. Patients were assigned to consume either pickle juice or tap water at the onset of a cramp, and outcomes were measured over a period of 28 days using a visual analog scale. The study found that pickle juice significantly reduced cramp severity compared to water, without affecting sleep quality or overall HRQoL [67]. Given that the mechanism of action is related to the acid in the brine interacting with an oropharyngeal receptor, in regions without kosher or dill pickles, one can consider trying other acidic liquids, such as vinegar.
- Baclofen: a gamma-aminobutyric acid agonist known for its muscle relaxant properties, has also been proposed as a potential pharmacological therapy for the treatment of muscle cramps. Data are limited regarding its effectiveness in trials. Baclofen can be started at a dosage of 10 mg daily, with the possibility of increasing the dose weekly as deemed necessary, up to a maximum dosage of 30 mg per day, divided into three doses [4]. Possible side effects include symptoms, such as confusion, dizziness, sedation, and gastrointestinal disturbances, such as nausea and vomiting [4]. Baclofen must not be used among patients with end-stage renal disease.
- Taurine: In addition, incorporating taurine into the treatment regimen of individuals experiencing muscle spasms associated with CLD may be a safe and effective intervention. The recommended dosage for taurine is 2–3 g per day [4, 68]. In a controlled trial involving 49 individuals with CLD, the efficacy of taurine in the treatment of muscle cramps was evaluated. The study found that 2 g of taurine per day resulted in a statistically significant reduction in the frequency, duration, and severity of cramps compared to a placebo, with no adverse effects observed [69]. The recommended dosage for taurine is 2–3 g per day.

We have also studied the role of nighttime stretching and meditation to reduce cramp severity. Both modalities were associated with improved cramp severity and sleep quality in a randomized trial called RELAX [70].

### Falls

Falls represent a significant complication in patients with cirrhosis, leading to diminished HRQoL, increased risk of mortality, and disability [71, 72]. While the incidence of falls among those with cirrhosis can vary based on a range of factors, it is generally observed that nearly half of the individuals diagnosed with this condition may experience such episodes [41, 71].

The underlying factors contributing to falls in people with cirrhosis are complex and varied and are closely related to the complications of the disease itself. In particular, cognitive impairment is a prominent factor [73]. Cirrhosis can precipitate HE, a condition that undermines critical cognitive faculties, including attention, reaction time, and executive function, all of which substantially heighten the risk of falls [73]. It is crucial to acknowledge that this heightened risk is not exclusive to a specific subset of patients with HE. It extends to individuals with both overt and covert forms of the disease [71, 74]. A retrospective study aimed to assess the link between minimal hepatic encephalopathy (MHE) and fall incidence in cirrhotic patients, including 130 outpatients and 43 controls. It was observed that 34.6% of patients with cirrhosis had MHE, and among them, 40% reported falls, a significantly higher rate compared to the 12.9% without MHE and akin to the control group at 11.6%. Additionally, those with MHE had a greater need for primary healthcare services (8.8% vs. 0%, *P *=* *0.004) and hospitalization (6.6% vs. 2.3%, *P *=* *0.34) due to falls compared to those without MHE. Notably, the 21 patients on psychoactive drugs demonstrated a more pronounced correlation between MHE and falls [74].

In addition to cognitive difficulties, sarcopenia contributes to the instability that leads to falls. The muscle strength, particularly in the lower extremities and core, is crucial for maintaining balance and for the rapid corrective actions required to prevent a fall when balance is lost. Weak muscles cannot produce the necessary force quickly enough to adjust the body’s position and prevent a fall [73]. Among the various risk factors for falls, a history of previous falls is the strongest predictor [71]. In addition, low blood sodium levels, decreased the number of chair-stands, lower Short-Form 8 (SF-8) scores, decreased albumin levels, dependence on assistance with activities of daily living, and use of medications, such as antidepressants, gabapentin, benzodiazepines, and opioids, all contribute to this increased risk [71]. In a prospective study of 299 patients with compensated Child-Pugh A-B cirrhosis and portal hypertension, but without prior HE, it was found that falls are a common and severe complication. Over a median follow-up of 1,003 days, 47% of patients experienced falls, with 13% suffering injuries. A predictive model for falls, the FallSSS score was validated for predicting the risk of falls. This model includes a history of previous falls, chair-stands, sodium levels, and the SF-8 health survey as predictors. These components were selected through a rigorous multivariable analysis and validated using cross-validation methods. In terms of performance, the FallSSS model showed a strong discriminative ability with an area under the receiver operating characteristic curve (AUROC) of 0.79 at 6 months and 0.81 at 12 months for predicting injurious falls. This was substantially better than the model for end-stage liver disease-sodium (MELD-Na), which had an AUROC of only 0.57 at the same time points. The model not only provided high predictive accuracy but also demonstrated strong associations with mortality, underscoring its clinical relevance in identifying cirrhosis patients at risk of falls for timely interventions [71].

The key strategies by which one can prevent falls are the elimination of zolpidem when possible [75], enrolling the patient in an exercise program proven to prevent falls, such as Tai-Chi [76], and treating cognitive dysfunction. An ongoing trial—LiveSMART—is evaluating the effectiveness of lactulose and tai-chi for the prevention of falls.

## General supportive care

For many symptoms, general supportive care should be reinforced. Two areas deserve specific attention. First, patients with cirrhosis must meet nutritional goals, such as a protein intake of 1 g/kg actual body weight and 30–40 kCal/kg each day with a nighttime snack. Muscle support and avoidance of catabolism is important for treatment of hepatic encephalopathy, which can impair coping and cause falls and could potentially help with muscle injury/cramps [77–80]. Such advice can be provided remotely or in person or by leveraging caregivers [79, 81]. Second, patient caregivers are desperate for guidance [82], should be seen as integral members of the care team, and should be assessed for burnout and financial duress and referred appropriately [83].

## Conclusions

The symptoms burden of CLD goes far beyond traditional complications and requires a comprehensive approach that encompasses both physical and psychological well-being. The interrelated and complex nature of symptoms, such as fatigue, pruritus, muscle cramps, SD, and falls, not only reduces HRQoL but may also contribute to a high risk of mortality. It is critical for healthcare providers to implement a holistic and compassionate approach that is based on the latest evidence while addressing the particular needs of their patients. Improvements in pharmacological interventions, coupled with refined screening and diagnostic methods and enhanced supportive care, will significantly improve patient outcomes and quality of life. Moreover, recognizing and prioritizing the wide range of symptoms associated with CLD is essential to significantly reducing symptom burden and improving the lives of those living with this complex disease.

## Authors’ Contributions

Each author contributed to the drafting of the manuscript.
